# Local psychiatric beds appear to decrease the use of involuntary admission: a case-registry study

**DOI:** 10.1186/1472-6963-14-64

**Published:** 2014-02-10

**Authors:** Lars Henrik Myklebust, Knut Sørgaard, Rolf Wynn

**Affiliations:** 1 Psychiatric Research Centre of Northern Norway, Nordland Hospital Trust, Bodø N-8092, Norway; 2 Department of Clinical Medicine, University of Tromsø, Tromsø N-9037, Norway; 3 Division of Addictions and Specialized Psychiatric Services, University Hospital of North Norway, Tromsø N-9291, Norway

**Keywords:** Involuntary admission, Health service, Social psychiatry

## Abstract

**Background:**

Studies on the effect of organizational factors on the involuntary admission of psychiatric patients have been few and yielded inconclusive results. The objective was to examine the importance of type of service-system, level of care, length of inpatient stay, gender, age, and diagnosis on rates of involuntary admission, by comparing one deinstitutionalized and one locally institutionalized service-system, in a naturalistic experiment.

**Methods:**

5538 admissions to two specialist psychiatric service-areas in North Norway were studied, covering a four-year period (2003-2006). The importance of various predictors on involuntary admission were analyzed in a logistic regression model.

**Results:**

Involuntary admission to the services was associated with the diagnosis of psychosis, male sex, being referred to inpatient treatment, as well as type of service-system. Patients from the deinstitutionalized system were more likely to be involuntarily admitted.

**Conclusions:**

Several factors predicted involuntary status, including male sex, the diagnosis of psychosis, and type of service-system. The results suggests that having psychiatric beds available locally may be more favourable than a traditional deinstitutionalized service system with local outpatient clinics and central mental hospitals, with respect to the use of involuntary admission.

## Background

The use of involuntary admission and other types of coercion in psychiatric care involves ethical dilemmas and may result in uncertain clinical outcomes [1-4]. Prior research has suggested that a range of factors may be important with respect to the use of involuntary admission and coercion in general, including sex, age, employment status, poverty, perceived dangerousness, and attitudes [5-11].

However, little is known about the how the organization of services may affect the use of involuntary admission, although a general increase has been seen in many countries following the area of deinstitutionalization of psychiatric care [12-15]. Some studies have suggested that reducing the number of hospital beds has led to an increase in the proportion of patients subjected to involuntary admission [16]. Other have suggested that changes in rates of involuntary admission over time more likely can be attributed to a broad set of factors, including changing legal frameworks, varying administrative routines, and differences in quality standards of treatment [17,18].

The rate of involuntary admission in Norway is among the highest in Europe [19]. Recent Norwegian studies have suggested that organizational factors may be important with respect to the use of coercion in general [20,21], but little is known about the importance of organizational factors to involuntary admission in Norway [10,11].

In the present study, we compared two demographically similar areas with differently organized services in a naturalistic experiment. The aim was to examine how a range of factors, including the organization of mental health services, affected rates of involuntary admission.

## Methods

According to Norwegian legislation [22], the involuntary admission of patients may be carried out when the patient is (or is suspected to be) ‘seriously mentally ill’ (in Norway this is a legal term which usually means that the patient suffers from a psychotic disorder) and when the patient is a danger to himself/herself or others and/or there is a need to admit the patient in order to secure that the patient gets the required treatment. GPs refer patients to admission, and may prefer to do so involuntarily when the patient does not consent but the GP believes that the patient meets the criteria for involuntary admission. The actual decision to treat a patient involuntarily is made by a psychiatrist or by a clinical psychologist at the institution responsible for the treatment (District Psychiatric Centre (DPC) or Central Mental Hospital (CMH)) within 24 hours of admission [22,23]. In the present study, all involuntary admissions took place at the Central Mental Hospital in Bodø, while a very small number of patients were treated as involuntary outpatients at the DPCs. However, most patients were treated voluntarily. The voluntary admission of an inpatient is ideally based on a joint understanding between the patient, the referring GP, and the providers at the receiving institution, that this is the best choice of treatment. Nevertheless, some patients that have been admitted legally voluntarily may feel that they have been pressured and not had a real choice, and some that have been admitted legally involuntarily may actually have accepted to be admitted [24-26]. The present study focuses on legal status only, as we have not had access to data comprising patients’ experiences with the admission process.

In Norway, the organization of psychiatric services differs somewhat between geographical areas. The DPCs, which provide the major part of all psychiatric services locally often differ in their organization of services. While some rely on several types of outpatient services locally in combination with inpatient services at larger centralized regional hospitals, other have beds available at small local psychiatric institutions [27,28]. The organizational differences may be of particular importance to the issue of involuntary admission, as in the study areas, only the Central Mental Hospital in Bodø admits inpatients involuntarily. The organization of the local DPCs and the degree to which they utilize services at the CMH may therefore affect the level of involuntary admission in the two areas.

The present study compares the neighbouring DPCs of Vesterålen and Lofoten, located in the County of Nordland, North Norway. The catchment areas’ characteristics strongly resemble each other structurally and demographically. The areas are characterised by small towns and communities along the coast. The majority of people work in fisheries, agriculture, tourism, small-scale industry, and public service. Communications to the county capital of Bodø are good, and local administrative institutions and educational facilities are in line with modern Norwegian standards. The population is very similar in the two areas in terms of the distribution of gender, age groups, and educational levels [29].

We also examined the epidemiological characteristics of the two areas by the use of publicly available statistics on living-conditions and demography. A ‘Care Need Index’ (CNI) was calculated, and weighted for size of the populations of the two catchment areas [30,31]. The estimated needs were remarkably similar, as the index of Lofoten was only slightly higher (45.4/42.2 = 1.07) than that of Vesterålen (54.6/57.8 = 0.94). To further verify this, we compared the rate of persons on disability pension with psychiatric diagnoses in the two areas, which turned out to be almost identical. Tables 1 and 2 give an overview of the characteristics of the catchment areas.

**Table 1:** Characteristics of the two catchment areas

	**Vesterålen**	**Lofoten**
Total number of inhabitants^1^	30 465	22 469
Inhabitants aged 18-65^1^	18 212 (59.7%)	12 734 (56.7%)
Cities^2^	2	2
Airports^2^	1	1
Larger harbours^2^	2	2
Travel time by air to County Capital (CMH) (in minutes)^3^	30	25
Share of CNI by catchment area size (%)^4^	54.6/57.8	45.4/42.2
Persons on disability (no/1000 inhab.)^4^	617 (20.2/1000)	447 (19.9/1000)

^1^By Statistics Norway (year of 2005) [29].

^2^Norwegian Mapping Authority [54].

^3^Widerøes Flyselskap A/S (Airline company) [55].

^4^Norwegian Labour and Welfare Administration [56].

**Table 2:** Age distribution in the two catchment areas

	**Vesterålen**	**Vesterålen**	**Lofoten**	**Lofoten**
	**Male**	**Female**	**Male**	**Female**
Young	2082 (11.4%)	1899 (10.4%)	1641 (12.2%)	1474 (11.0%)
Middle aged	4111 (22.6%)	3989 (22.0%)	3029 (22.6%)	2916 (21.7%)
Elderly	3147 (17.3%)	2984 (16.4%)	2285 (17.0%)	2072 (15.4%)
Sum	9340 (51.3%)	8872 (48.7%)	6955 (51.8%)	6462 (48.2%)

Data from Statistics Norway (year of 2005) [29].

**Figure 1:**
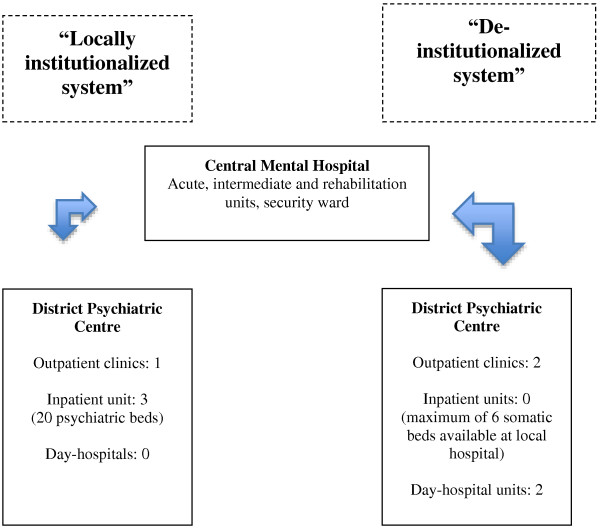
Illustration of the psychiatric service-systems in the sectors of Vesterålen and of Lofoten, County of Nordland, Norway.

The psychiatric services on the other hand, are very differently organized [27,32]. In Vesterålen, 70% of all inpatient admissions are at the local DPC, while the rest are at the Central Mental Hospital (CMH) in Bodø. In Lofoten, the majority is provided at the CMH and only 10% locally at the DPC (i.e. actually in beds located at a local somatic hospital, see Figure 1). Thus, more outpatient clinics and day-hospital units are provided in Lofoten. The rate of outpatient clinicians differs with 2.0 per 1000 inhabitants in Lofoten versus only 1.1 per 1000 inhabitants in Vesterålen. Consequently, the two systems may be termed a ‘deinstitutionalized system’ (i.e. in Lofoten) versus a ‘locally institutionalized system’ (i.e. in Vesterålen). For both systems, all involuntary admissions of inpatients take place at the CMH in Bodø.

In a previous study, we found that the rates of hospitalization were remarkably alike between the systems, with a population rate of 7.7 inpatients per 1000 (Vesterålen) vs. 8.4 per 1000 (Lofoten), and a bed-utilization rate of about 1 per 1000 inhabitants in both areas [27]. This suggests that the use of inpatient treatment is quite similar in the two service-systems.

Drawing on the routine case-registries of the services, 5538 individual treatment-episodes in the four-year period 2003-2006 were identified. These represent all admissions to the psychiatric specialist services in the two areas, both at the local level of the DPCs and at the CMH. The registry contains demographic variables, service-variables, and clinical variables. All clinical variables in the registries were registered by clinical staff during individual treatment courses. The use of diagnostic tools and procedures in the services was not systematic. Diagnoses of inpatients were often discussed and set in joint meetings with psychiatrists, clinical psychologists, and specialist nurses. Only 9 patients were treated in both areas, and less than .5% were outside residents. These were omitted from further analyses. Missing data were collected from medical records when possible.

Involuntary admissions were analysed by studying the following variables: A) gender, age and home-address; B) the level of care (outpatient/inpatient) and the volume of treatment (length of inpatient stay and number of outpatient consultations/day-hospital use); and C) diagnoses (according to the ICD-system [33]).

In order to obtain adequate sizes for analysis, we collapsed less frequent diagnoses into eight broader categories: 1) Psychiatric observation (ICD-10 Z03.2, Z04), 2) Substance abuse (F10-19), 3) Psychotic disorders, including affective psychoses, excluding substance related psychoses and organic psychotic disorders (F20-29 and F30.2, 31.2, 31.5, 32.3, 33.3), 4) Affective disorders, excluding affective psychoses (F30-39, except 30.2, 31.2, 31.5, 32.3, 33.3), 5), Anxiety disorders (F40-48), 6) Anorexia and other eating disorders (F50), 7) Personality disorders (F60-62), and 8) ‘Other’ (including dementia (F00-04), organic psychoses and deliria (F05-09), hyperkinetic disorders (F90) and ‘social problems’ (Z64-65).

Uni-variate analyses of differences were performed with Chi-square tests, t-tests, and Mann-Witney U-tests. A multivariable logistic regression analysis was performed with all variables as predictors to control for possible interactions, and with legal status at admission as the dependent variable (involuntary/voluntary). The predictors were entered in a stepwise manner, following the Forward LR method in SPSS (‘Observation’ was used as contrast for diagnoses). To increase readability, variables that did not reach the level of significance were omitted from the resulting table, with the exception of age.

The present study was approved by the Regional Medical Ethics Committee, the Norwegian Data Protection Agency, and the Norwegian Directorate for Health.

## Results

Table 3 compares the overall (inpatients and outpatients) population in the two systems by univariate analysis. The results show that there are some differences in age, gender, and diagnostic categories.

**Table 3:** Patient and treatment characteristics of all admissions in a locally institutionalized versus a deinstitutionalized system of mental health services

**Service system**		**Deinstitutionalized**	**Locally institutionalized**
**N**		**2022**	**3316**
**Age** (mean)		38.0 (Sd = 13.3)**	39.5(Sd = 13.1)**
**Gender**	Female	1118 (55.3%)**	1988 (60.0%)**
	Male	904 (44.7%)**	1328 (40.0%)**
**Diagnosis**	Observation	339 (16.8%)**	917 (27.7%)**
	Substance abuse	255 (12.6%)**	135 (4.1%)**
	Psychosis	218 (10.8%)**	535 (16.1%)**
	Affective	400 (19.8%)*	759 (22.9%)*
	Anxiety	444 (22.0%)	695 (21.0%)
	Anorexia n.	66 (3.3%)**	56 (1.7%)**
	Personality d.	137 (6.8%)**	121 (3.6%)**
	Others	163 (8.1%)**	98 (3.0%)**
**Level of care**	Inpatient	758 (37.5%)	1174 (35.4%)
	Outpatient	1264 (62.5%)	2142 (64.6%)
**Involuntary admission**		113 (5.6%)*	131 (4.0%)*
**Treatment volume** (median)	Inpatient stay (length of-)	5**	8**
	Outpatient consultations	3**	2**

4-year registered prevalence sample (2003-2006), N = 5338.

*P < 0.05, **P < 0.01 when service-models are compared.

Most relevant for the present study are the differences in the variables regarding involuntary admission. The deinstitutionalized system had a significantly higher rate of involuntary admission than the other system (5.6% vs. 4.0%), see Table 3.

In order to control for possible interactions or differences in populations, a multivariable analysis was performed. The model was statistically significant (N = 5338, Chi-square 498.541, d.f. = 13, p < .000), indicating that it could distinguish between legal status at admission (i.e. voluntary or involuntary). Log-likelihood was 1483.825, and the model explained between 9.0% (Cox & Snell R Square) and 28.7% (Nagelkerke R Square) of the variance in legal status at admission. The results are displayed in Table 4.

**Table 4:** Logistic regression model of involuntary admissions (n/y) in a local-bed versus a central-bed system of mental health services

**Variable**	**B**	**Sig.**	**EXP(B)**	**95% C.I. for EXP(B)**	
**Gender** (F = 0, M = 1)	.431	.003	1.539	1.157	2.048
**Age**	-.004	.422	.996	.986	1.006
**Psychosis**	.956	.003	2.600	1.372	4.930
**Service-system** (Local-bed = 0, Central-bed = 1)	.415	.005	1.515	1.133	2.026
**Level of Care** (Inpatient = 0, Outpatient = 1)	-2.948	.000	.052	.026	.106
Constant	-2.483	.000	.084		

County of Nordland, Norway, 4-year registered prevalence sample, N = 5338.

The diagnostic category of *Psychosis* emerged as the strongest predictor of involuntary admission, giving a confidence interval for EXP(B) of 1.372 – 4.930 (p = .003), controlling for all other factors in the model. This indicated that patients with psychosis-related disorders were more likely to be involuntarily admitted than patients with other diagnoses. Also, male patients were more likely to be involuntarily admitted than female patients. Notably, besides these known and individual predictors for involuntary admission, the most important predictor was *Service-system*, which had a confidence interval of 1.133 – 2.026 (p = .005). This indicated a higher risk for patients from the deinstitutionalized system of being involuntarily admitted.

## Discussion

The most important finding of this study was that the deinstitutionalized system without available local beds had the highest rate of involuntary admission. In addition, the present study also supported prior findings suggesting that male patients, patients suffering from psychotic disorders, and patients that are referred to inpatient stays, are more likely to be involuntarily admitted [10,34,35].

This study is one of very few in recent years that have studied whether the organisation of services is of importance to the use of involuntary admission. Some earlier studies have suggested that organisational and structural differences in psychiatric care may be of importance to rates of involuntary admission [36,37]. In our natural experiment, we studied two neighbouring and comparable catchment areas with different models of service-systems, one ‘deinstitutionalized’ and one ‘locally institutionalized’. We found that having psychiatric beds available locally appeared to decrease the use of involuntary admission. A higher proportion of involuntary admission was found in the system with beds located mainly at a Central Mental Hospital.

Continuity of care for severely ill patients has been an important topic in mental health services research [38,39]. Several studies have found a positive relationship between high levels of continuity of care and important outcomes such as improved quality of life, improved community functioning, lower severity of symptoms, and greater service satisfaction [40,41]. We believe one possible explanation for the importance of service-system on the rate of involuntary admission may be that the proximity and local control of psychiatric beds and the integration of services lower the threshold for inpatient admission and thereby allow patients to be readily admitted before the condition becomes too grave [32,42-44]. The finding that having psychiatric beds available locally may be conducive to a lower rate of involuntary admission could therefore in part be due to a higher integration of services in the locally institutionalized system. This theory is in accordance with a prior study, where it was found that the system with locally available beds had a better integration of inpatient and outpatient services, and that clinicians in the service-system with beds available locally were better able to follow up patients that made use of different types of services [32].

Another possible explanation may be that the fact that many of the inpatients in the deinstitutionalized system are treated in a Central Mental Hospital may increase the risk of being subjected to different types of coercion, including involuntary admission [20,21]. The involuntary admission of inpatients only takes place at the Central Mental Hospital in our study and patients that are referred for inpatient stays at the DPCs in our study can only be treated voluntarily.

The present results may also be in accordance with the longstanding notion of supply–induced demand [45,46]. The higher rate of involuntary admission in the deinstitutionalized system may be related to the fact that most inpatients in this system are assessed and treated by psychiatrists and clinical psychologists that have wards available that may be used for involuntary treatment. Hence, clinical decisions may be influenced by the possibilities and limitations inherent in the organisation of services.

Another possible explanation may be that, in the service area without available local psychiatric beds, the GPs and local psychiatric specialists more often prefer to refer patients involuntarily strategically (i.e. to the Central Mental Hospital), as there could be a perception that involuntary patients are more likely to be accepted for admission.

### Strengths and limitations

One weakness of this study is that the data concerns admissions to psychiatric specialist services rather than individual patients. This may introduce a bias as some patients may have been admitted to the services several times, and some might have been referred both to inpatient services and outpatient services. On the other hand, the data concern more than 5000 admissions, which is a relatively high number and a strength in case-register studies [47]. Most prior studies of involuntary admission and other types of coercion have been carried out on relatively small data-sets. Moreover, we lack data on some variables that could be of importance to the use of involuntary admission, including degree of disability, financial status, employment, educational level, ethnicity, attitudes of doctors, caregivers and patients, etc. [5,6,8,10,23,34,48-51]. The model predicts between 9.0% (Cox & Snell R Square) and 28.7% (Nagelkerke R Square) of the variance in legal status at admission. This suggests that the model is relatively strong, even though we lack some variables that could be of importance to the prediction of legal status at admission.

The diagnoses used in this study are based on routinely collected data. There might be limitations in accuracy and standardization of information collection that may impact the quality of the data. The observed differences in diagnoses (Table 3) could in part be attributable to differences in the use of diagnostic tools and/or registration procedures in the two areas. As most patients had received only one diagnosis, we utilized this main diagnosis in the analyses. This could be a weakness, as some patients are likely to fulfill criteria for more than one diagnosis. For instance, the amount of patients with a substance use related diagnosis is lower in this study than in other Norwegian studies that have utilized other designs [52,53]. There is also a relatively high proportion of patients in the ‘Observation’ category. Some of these patients have been assessed and not fulfilled criteria for a psychiatric diagnosis. Others are patients that are being examined and that have not yet received a diagnosis. There is some missing data in the case-registry, this is most pronounced for certain variables such as level of functioning and for data entered by staff working in the DPCs. We believe this may be related to less-than-ideal registration procedures possibly due to lack of awareness or training.

One of the major advantages of the study is the control over the patient-population. There are no private local providers, and availability of services outside the catchment areas is limited due to considerable geographical distance [54,55]. Thus, there is very little cross-boundary service provision. Consequently, the case-registries sample all psychiatric patients in the systems. When the two areas are compared (see Table 3), we see that the difference in age and gender is small, but this may indicate that the patient populations nevertheless differ somewhat. This probably reflects the differences in organization, where the deinstitutionalized system selects more outpatients into treatment. Also, the significant differences found for amount of treatment was expected given this difference in organisation of services. Thus, there might be a bias in the selection of patients into psychiatric care in the two service-systems. The case-register at hand cannot satisfactorily resolve this issue [47]. A future study involving a closer assessment of the patients in levels of disability and standardized procedures for diagnostic practice could reveal whether one of the systems selects more disordered or disabled persons into treatment than the other [56].

However, as the two service areas compared in this study are very similar demographically and in other respects – we believe that it is unlikely that the population in the two areas differ substantially. Hence, we believe that this natural experiment lends support to the idea that organizational factors may affect the use of involuntary admission, either through the selection of persons into treatment or through clinical decisions made during treatment.

## Conclusions

Factors such as diagnosis, gender, type of service system, and level of care are predictors of importance to patients’ legal status. Having psychiatric beds available locally may be a more favourable type of psychiatric service organization with respect to minimizing the use of involuntary admission.

## Competing interests

The authors declare that they have no competing interests.

## Authors’ contributions

LHM designed the study, collected the data, analysed the data, drafted the manuscript, revised the manuscript and approved the final manuscript. KS designed the study, analysed the data, revised the manuscript and approved the final manuscript. RW analysed the data, drafted the manuscript, revised the manuscript and approved the final manuscript. All authors read and approved the final manuscript.

## Pre-publication history

The pre-publication history for this paper can be accessed here:

http://www.biomedcentral.com/1472-6963/14/64/prepub
